# Relation between Selected Sleep Parameters, Depression, Anti-Tumor Necrosis Factor Therapy, and the Brain-Derived Neurotrophic Factor Pathway in Inflammatory Bowel Disease

**DOI:** 10.3390/metabo13030450

**Published:** 2023-03-19

**Authors:** Marcin Sochal, Marta Ditmer, Agata Binienda, Agata Gabryelska, Piotr Białasiewicz, Renata Talar-Wojnarowska, Jakub Fichna, Ewa Małecka-Wojciesko

**Affiliations:** 1 Department of Sleep Medicine and Metabolic Disorders, Medical University of Lodz, 90-419 Lodz, Poland; 2 Department of Biochemistry, Medical University of Lodz, 90-419 Lodz, Poland; 3 Department of Digestive Tract Diseases, Medical University of Lodz, 92-215 Lodz, Poland

**Keywords:** IBD, BDNF, anti-TNF therapy, sleep, depression

## Abstract

Inflammatory bowel disease (IBD) patients often have sleep and mood disorders. Brain-derived neurotrophic factor (BDNF) and proBDNF were shown to modulate interactions between the central nervous system and the gastrointestinal tract, possibly contributing to psychological issues. Anti-tumor necrosis factor (TNF) therapy in IBD can alter BDNF expression and further affect the brain–gut axis. Eighty IBD patients and 44 healthy controls (HCs) were enrolled and divided into subsets based on disease activity and condition (ulcerative colitis (UC)/Crohn’s disease (CD)). Questionnaires evaluating sleep parameters and depression as well as venous blood were collected. The IBD group had a lower expression of BDNF mRNA, but higher proBDNF and BDNF protein concentration than HCs. The UC group had a higher BDNF protein concentration than the CD. BDNF protein was positively correlated to sleep efficiency in the IBD group. Depression severity was associated positively with BDNF mRNA and negatively with BDNF protein in the remission group. Anti-TNF therapy enhanced BDNF mRNA expression. The BDNF pathway might be disturbed in IBD, linking it to sleep disorders and depression. Systemic inflammation could be the main cause of this disruption. BDNF mRNA is a more reliable parameter than protein due to numerous post-translational modifications.

## 1. Introduction

Inflammatory bowel diseases (IBDs) are a group of inflammatory conditions with immune background affecting the gastrointestinal tract. Two canonical types of IBD are Crohn’s disease (CD) and ulcerative colitis (UC), which differ in disease manifestations, expansion area, endoscopic and pathological findings, as well as treatment [1,2]. They tend to have a waxing and waning course, like many other chronic inflammatory conditions. IBD, apart from physical, carries a psychological burden: studies have shown that this group of patients tends to be more frequently afflicted with sleep disturbances and depression than the general population [3,4]. What is more, anxiety and depression could be related to more severe disease course [5].

Mechanisms mutually connecting IBD and accompanying psychological issues remain elusive. It appears that neurotrophins (NTs), brain-derived neurotrophic factor (BDNF) in particular, have an important role in the pathophysiology of both IBD and accompanying psychological conditions. NTs form a group of similarly structured proteins, which maintain the functioning of the central nervous system (CNS). BDNF is important for neuronal excitability and synaptic plasticity, as well as protection against apoptosis. It exerts its effect by activating the tropomyosin kinase B receptor (TrkB) pathway. BDNF’s premature form, proBDNF, is also metabolically active, albeit in that it has, in many aspects, opposite effects to TrkB, stimulating apoptosis, synaptic elimination, and decreasing synaptic strength [6,7]. Its receptor is p75 neurotrophin receptor (p75NTR) [8]. What is important is that, even though the majority of BDNF is produced within the CNS, it can cross the blood–brain barrier. Thus, concentration of this protein, but not mRNA, in the periphery might reflect that within the CNS.

BDNF has been found to have a mutual connection with sleep structure [9]. A decrease in BDNF expression resulting from polymorphism Val66Met was related to lower sleep intensity, slow-wave sleep duration, and alterations of slow-wave activity (prolonged onset and dissolution) [10]. Low BDNF was also associated with a reduction in N3 and rapid eye movement stage [11]. On the other hand, insomnia decreases BDNF levels, while an acute form of sleep deprivation increases them. This diverse response is most likely attributable to differences in stress response to such adversity [12]. BDNF has also been extensively studied in the context of mental health; its decreased levels were observed in psychological disorders, such as dementia or depression, although those changes are not specific [13]. Moreover, it appears to mediate the effects of antidepressants [13]. In individuals suffering from major depressive disorder, an increase in BDNF was associated with longer non-rapid eye movement sleep duration as well as slow-wave activity [14].

NTs’ receptors are prevalent in the gastrointestinal tract: both TrkB and p75NTR are present in endocrine cells, submucosal and myenteric plexus, as well as enteric glia [15,16,17,18]. Impairment of the enteric glia, caused by BDNF deficiency, might lead to malfunctioning of the intestinal barrier, which may contribute to IBD pathogenesis [18,19]. Blockade of the TrkB-PLC/IP3 pathway in experimental colitis in mice had detrimental consequences [20]. It increased the enterocyte apoptosis rate and shifted the cytokine profile towards more pro-inflammatory, which resulted in a more severe disease course, confirmed by biopsy findings [20].

BDNF and tumor necrosis factor (TNF) expression could be connected. Metalloproteinase 9, an enzyme important in the process of BDNF production, can be stimulated by TNF [21,22]. As Prakesh et al. have demonstrated, BDNF and TNF might work in a synergistic manner, enhancing the contractile response to agonists of smooth airway muscle cells [23]. In a study on rat colon smooth muscle cells, application of TNF and interleukin 18 promoted BDNF production [24]. Therapy with anti-TNF agents has long been successfully applied in IBD patients [25,26]. However, its relation to BDNF level in this group is scarcely evaluated. Effects of such treatment in other conditions vary; data obtained from IBD patients would help to broaden the knowledge on this subject.

This study aimed to investigate the selected neurotrophin-related mechanisms of sleep and mood disorders in IBD, as well as evaluate the influence of anti-TNF therapy on mentioned processes.

It is a continuation of our previous project on patients with CD [27]. However, the scope of the current study was extended in comparison to the original; we included analysis of the BDNF mRNA expression and BDNF and proBDNF protein serum concentration in order to obtain a more complex insight into the role of the BDNF pathway in IBD, considering modifications that might preclude or promote protein production from mRNA transcript. This is a novelty, as most studies, such as the pilot, focused only on BDNF concentration.

## 2. Materials and Methods

### 2.1. Recruitment and Patient Eligibility

A group of 80 IBD patients was enrolled in the study together with 44 healthy controls (HCs), matched for body mass index, sex, and age. All participants were recruited at the Department of Digestive Tract Diseases (Medical University of Lodz, Lodz, Poland). The sample size was not calculated prior to the study due to the limited amount of available literature on the subject, especially regarding the role of proBDNF in IBD. Applied inclusion criteria were as follows: UC or CD diagnosis (according to the histopathological, clinical, and endoscopic criteria), signing a written informed consent to participate in the study, age 18–65 years old. Exclusion criteria included any other chronic non-inflammatory disease (hypertension, type II diabetes, hypercholesterolemia, etc.), psychological disorders and/or use of psychoactive substances in the anamnesis, malignancy other than basal cell carcinoma, laparotomies and/or laparoscopic surgery in the preceding 6 months.

The study protocol has received approval from The Bioethical Committee of the Medical University of Lodz, Poland (number: KE/1139/20).

### 2.2. Acquisition of Material

Participants filled out the following questionnaire forms: Athens Insomnia Scale (AIS), Pittsburgh Sleep Quality Index (PSQI), Epworth Sleepiness Scale (ESS), Beck Depression Inventory (BDI); Harvey–Bradshaw Index (HBI) or partial Mayo score, depending on the IBD type, were completed by a trained physician [28,29,30,31,32]. Samples of peripheral venous blood, at the volume of 9 mL, were collected between 9.00 and 11.00 a.m.; in the group qualified for anti-TNF therapy, blood was drawn twice, at the baseline and after 14 weeks of induction therapy.

### 2.3. Assessment of Disease Severity and Psychological Variables

Assessment of the disease clinical disease severity was performed with the use of partial Mayo score or Harvey–Bradshaw Index (HBI) in UC and CD, respectively.

HBI involves evaluation of the following domains: well-being, the severity of abdominal pain, quantity of soft/liquid stools the previous day, complications (either extra or intra-abdominal, such as arthralgia, erythema nodosum, or a new fistula) [33]. A score of fewer than 5 points in individuals who are currently not taking steroid medications indicates CD remission [33]. Partial Mayo score comprises 3 domains: frequency of bowel movement, rectal bleeding, and physician’s global assessment [34]. Exacerbation was defined as a score of at least 2 points [34]. Participants who scored less than 2 points were assigned to the remission group.

For the sake of standardizing the outcomes, only one trained researcher evaluated the disease activity in the study’s participants.

Sleep and mental health were assessed with the use of appropriate questionnaires. BDI is a questionnaire evaluating the severity of depression symptoms [28]. It comprises 21 items, each rated on a scale 0–3, 3 indicating the highest intensity of a given disease manifestation [28]. The subject can assess the severity and frequency of symptoms on a 4-point scale in each item [28]. Subjects who scored 11 points or above were considered to have pronounced depression symptoms [28]. AIS is an instrument applied in the diagnostic process of insomnia. It consists of 8 domains (e.g., daytime sleepiness, sleep duration) [29,30]. Subjects can grade the severity of symptoms on a scale 0–3. Scoring at least 5 points in this questionnaire suggests insomnia [29,30]. PSQI is a tool for the assessment of subjective sleep quality in general. The individual assesses a diverse range of domains associated with sleep quality, such as sleep continuity or difficulty in falling asleep [31]. Subjects who scored more than 5 points are considered to have poor sleep quality, which might require further clinical investigation [31]. ESS evaluates the severity of daytime sleepiness [32]. In this questionnaire, the individual estimates the probability of undesirable onset of sleep in 8 situations from daily life. Each item might be assigned 0–3 points [32]. A score above 10 indicates a pathological daytime sleepiness [32].

### 2.4. Anti-TNF Therapy

IBD participants (n = 26) in exacerbation, where indicated, were treated with anti-TNF medications (adalimumab or infliximab). The treatment protocol was in line with the guidelines of the European Crohn’s and Colitis Organization. Medications were administered as follows: infliximab was given intravenously (5 mg/kg body weight) at weeks 0, 2, 6, 14; adalimumab was injected subcutaneously at weeks 0, 2, and later every 2 weeks at a dose of 160, 80, and 40 mg, respectively, for each time point. Follow-up visits were conducted after 14 weeks and included collection of venous blood, questionnaires, and disease severity evaluation.

### 2.5. Evaluation of Gene Expression and Protein Concentration

RNA was isolated from peripheral blood mononuclear cells using the ‘TRIzol method’ with TRIzol (Invitrogen). RNA concentration and integrity number (RIN) was measured by Nanodrop Colibri Microvolume Spectrometer (Colibri microvolume spectrometer, Titertek Berthold, Pforzheim, Germany). Reverse transcription was conducted with an appropriate kit according to the manufacturer’s protocol (SuperScript IV First-Strand Synthesis System, Thermo Fisher Scientific Inc., Santa Clara, CA, USA). Reverse transcription was a 3-step procedure; assays annealing took place at 60 °C for 60 s. Reverse transcription and cDNA synthesis was performed with Biometra T-personal Thermocycler (Biometra, Gottingen, Germany).

Analysis of gene expressions was performed by quantitative real-time polymerase chain reaction (qPCR), for which nuclease-free water, cDNA, gene specific assays (TaqMan assays for: BDNF, TNF, reference gene: β-Actin), Master Mix TaqMan Universal were used. qPCR was performed by Rotor-GeneTM 3000 (Corbett Research, Corbett Life Science, Mortlake, Australia) two times per sample and reference; cycle threshold (CT) was determined for each sample. The outcomes were rendered as ΔCT and analyzed with the Livak formula [35]. 

Analysis of serum BDNF and proBDNF protein concentration was performed with the use of ELISA kit (Human BDNF ELISA Kit and Human proBDNF ELISA Kit, FineTest, Wuhan, China). For both proteins, the absorbance level was set at λ = 450 nm; measurements were taken with the absorbance reader (BioTek 800 TS, Agilent Technologies, Santa Clara, CA, USA).

Study procedures were performed in the Department of Biochemistry and the Department of Sleep Medicine and Metabolic Disorders, Medical University of Lodz.

### 2.6. Statistical Analysis

Statistica 13.1 PL (StatSoft, Tulsa, OK, USA) was used to conduct the statistical analysis. The level of statistical significance was set at *p* < 0.05. Shapiro–Wilk test was applied to classify the distribution of continuous variables as normal or non-normal. Mean and standard deviation or median and interquartile range (IQR—interquartile range, first–third quartile) was calculated for data with normal or non-normal distribution, respectively. The Mann–Whitney U or Wilcoxon test were used for the analysis of independent or dependent nonparametric variables. Parametric independent variables required the use of Student’s *t*-test. Spearman’s correlation test was performed to assess associations between variables. Pre-/post-therapy protein concentration and mRNA expression level ratios were also calculated.

## 3. Results

Baseline data characterizing the study’s participants are compiled in Table 1. 

The HC group did not significantly differ from the study group in terms of sex, age, BMI, smoking, or chronic non-inflammatory diseases; similarly, the exacerbation group matched the remission group in demographic and clinical parameters. There were also no differences between baseline TNF mRNA expression levels in IBD vs. HC or exacerbation vs. remission (*p* = 0.817 and *p* = 0.222, respectively).

**Figure 1:**
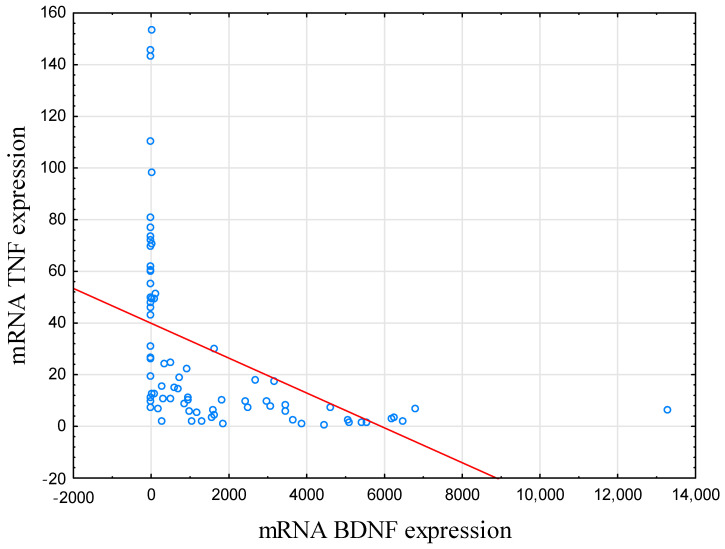
Correlation between mRNA BDNF and mRNA TNF expression. In IBD subjects, there was a negative correlation between BDNF and TNF mRNA expression level (r = −0.758, *p* < 0.001). Abbreviations: BDNF—Brain-Derived Neurotrophic Factor, TNF—Tumor Necrosis Factor.

BDNF mRNA expression level was negatively correlated with TNF mRNA in IBD subjects (r = −0.758, *p* < 0.001; Table 2; Figure 1). In HCs, this correlation was not significant (r = −0.285, *p* = 0.061).

**Figure 2:**
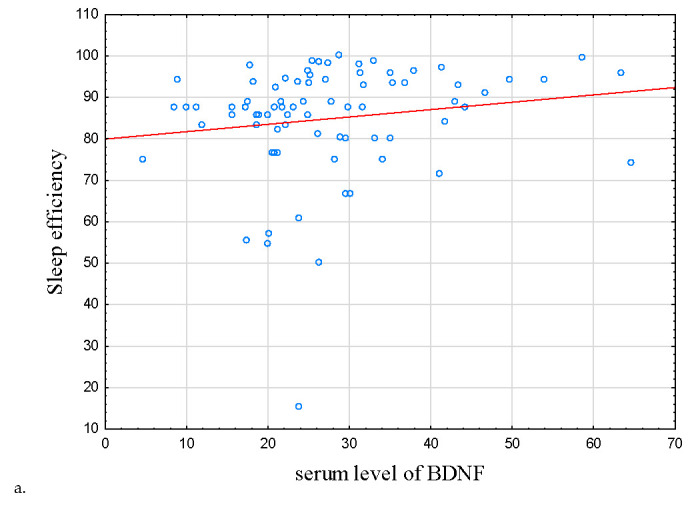

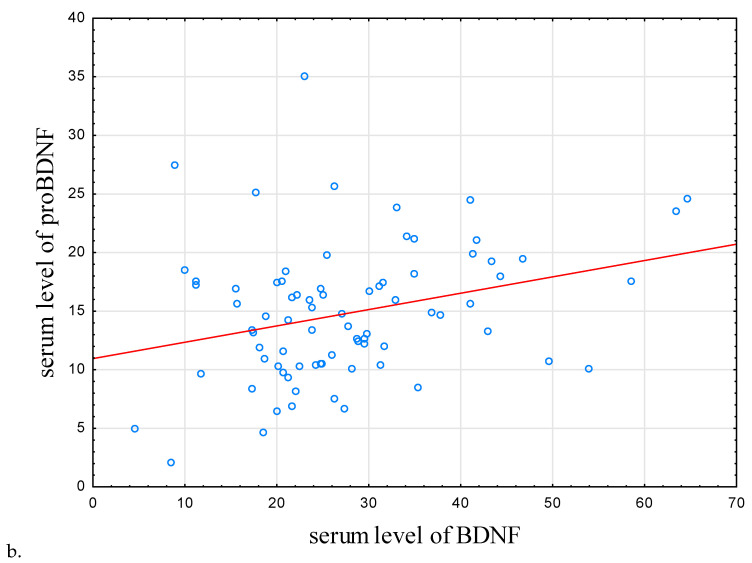
Correlations between sleep efficiency, serum level of proBDNF, and BDNF. (**a**) Correlation between serum level of BDNF and sleep efficiency. (**b**) Correlation between serum level of BDNF and proBDNF. In IBD subjects, there were positive correlations between sleep efficiency, serum level of proBDNF, and serum level of BDNF (r = 0.234, *p* = 0.037 and r = 0.292, *p* = 0.009), respectively. Abbreviations: BDNF—Brain-Derived Neurotrophic Factor, proBDNF—BDNF precursor.

It was observed that BDNF mRNA expression in HCs was higher than that in the IBD group (*p* = 0.008; Table 2). Counterintuitively, the concentration of BDNF and proBDNF proteins was higher in the IBD individuals compared to the control subjects (*p* = 0.008 and *p* < 0.001, respectively). In IBD individuals and HCs, proBDNF and BDNF protein concentration were positively correlated (r = 0.292, *p* = 0.009, r = 0.593, *p* < 0.001, respectively; Table 3; Figure 2b).

As for sleep parameters, there was a correlation between BDNF protein concentration with subjective sleep efficiency in the IBD group (r = 0.234, *p* = 0.037, Table 3 and Table 4; Figure 2a); this correlation did not reach statistical significance in HCs (r = −0.128, *p* = 0.409). 

Associations between other sleep variables (sleep latency, daytime sleepiness, sleep quality, total sleep time, insomnia symptoms) and studied proteins or mRNA levels did not reach statistical significance.

**Figure 3:**
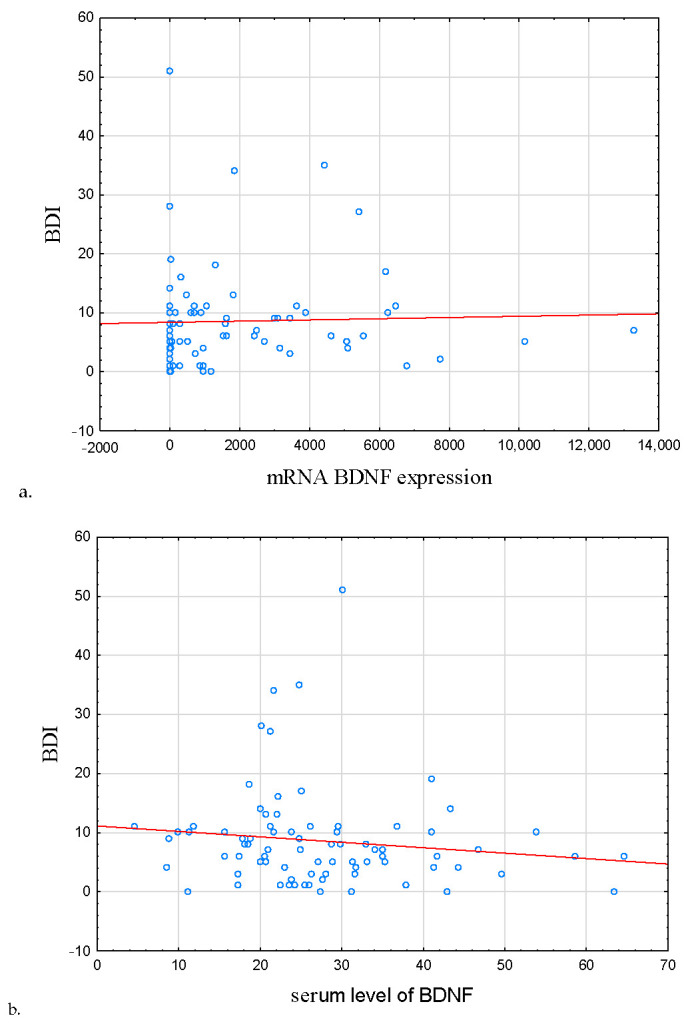
Correlation between BDNF mRNA expression, serum level of BDNF, and severity of depression symptoms. (**a**) Correlation between mRNA BDNF expression and BDI, (**b**) correlation serum level of BDNF and BDI. In IBD subjects in remission, the severity of depression symptoms was correlated positively with BDNF mRNA expression and negatively with BDNF protein concentration (r = 0.363, *p* = 0.038; r = −0.349, *p* = 0.046, respectively). Abbreviations: BDI—Beck’s Depression Inventory, BDNF—Brain-Derived Neurotrophic Factor.

In IBD subjects in remission, the severity of depression symptoms was correlated positively with BDNF mRNA expression and negatively with BDNF protein concentration (r = 0.363, *p* = 0.038; r = −0.349, *p* = 0.046, respectively; Table 4, Figure 3a,b, respectively). 

Additionally, in this group, BDI score was correlated positively with symptoms of insomnia, daytime sleepiness, and poor sleep quality, but negatively with sleep efficiency (r = 0.515, *p* < 0.001, r = 0.298, *p* = 0.007, r = 0.536, *p* < 0.001, r = −0.306, *p* = 0.006, respectively).

In HCs, the score of BDI was negatively correlated with both BDNF protein and mRNA; however, those associations were not significant (r = −0.125, *p* = 0.418, r = −0.068, *p* = 0.661).

The UC patients had a higher BDNF protein concentration than CD patients (*p* = 0.007; Table 2). However, no differences were noted regarding proBDNF protein level or BDNF mRNA expression. Nevertheless, the severity of depression symptoms was positively correlated with symptoms of insomnia, daytime sleepiness, poor sleep quality (r = 0.612, *p* < 0.001, r = 0.385, *p* = 0.010, r = 0.527, *p* < 0.001, respectively). Correlation between BDI and sleep efficiency was not significant (r = −0.110, *p* = 0.476).

No differences between IBD patients in exacerbation and remission were observed regarding protein concentration or mRNA level. Nevertheless, both subgroups had a higher concentration of BDNF protein and its precursor than HCs (*p* = 0.002, *p* = 0.043 and *p* < 0.001, *p* = 0.009 for BDNF and its precursor in exacerbation and remission, respectively; Table 2). Expression of BDNF mRNA was also decreased in both subsets, compared to HCs (*p* = 0.025 and *p* = 0.019 for exacerbation and remission, respectively). Correlations between HBI, partial Mayo score, and BDNF protein were not significant (r = −0.144, *p* = 0.325, r = −0.067, *p* = 0.720 correspondingly).

**Figure 4:**
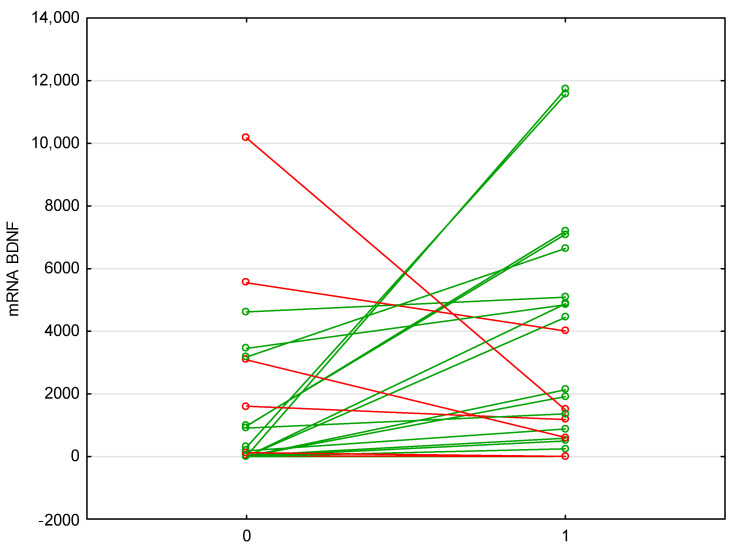
Changes in the expression of BDNF mRNA before and after biological therapy. Anti-TNF therapy with either infliximab or adalimumab increased the level of mRNA BDNF expression, while decreasing anti-TNF mRNA (*p* = 0.009, *p* = 0.045). Abbreviations: BDNF—Brain-Derived Neurotrophic Factor. Green color indicates an increase, red decrease.

Anti-TNF therapy with either infliximab or adalimumab increased the level of mRNA BDNF expression, while decreasing anti-TNF mRNA (*p* = 0.009, *p* = 0.045, respectively; Table 5; Figure 4). 

**Figure 5:**
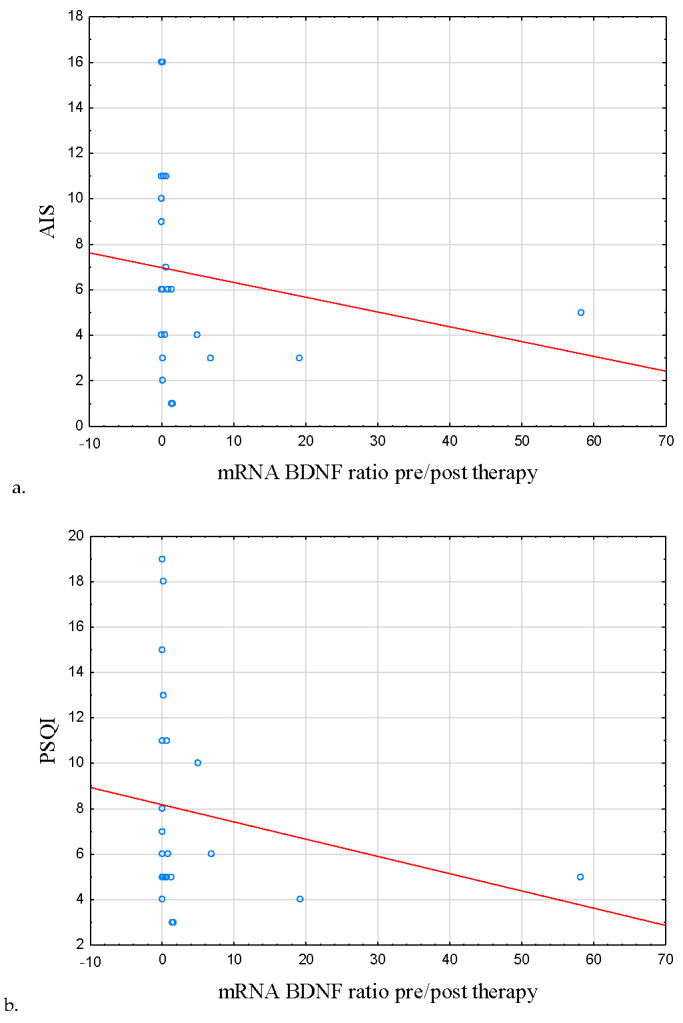

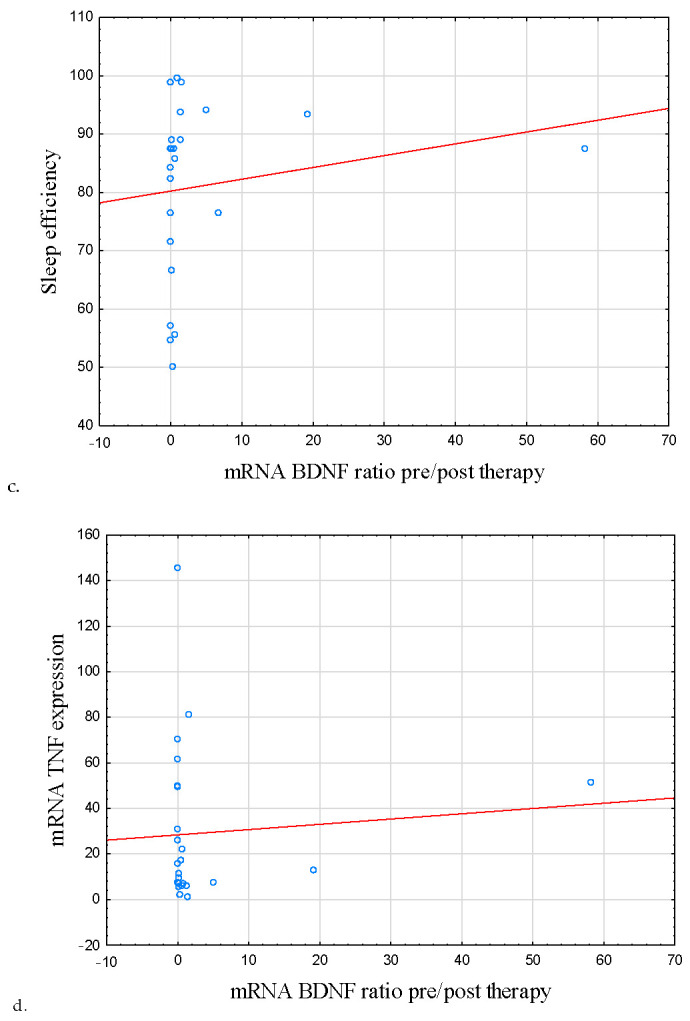
Correlation between symptoms of insomnia, poor sleep quality, sleep efficiency, mRNA TNF, and BDNF mRNA ratio pre/post therapy. (**a**) Correlation between AIS score and BDNF mRNA ratio pre/post therapy. (**b**) Correlation between PSQI score and BDNF mRNA ratio pre/post therapy. (**c**) Correlation between sleep efficiency and BDNF mRNA ratio pre/post therapy. (**d**) Correlation between TNF mRNA and BDNF mRNA ratio pre/post therapy. mRNA BDNF ratio pre/post therapy was negatively correlated with the severity of insomnia symptoms (AIS score) at the baseline (r = −0.409, *p* = 0.042) as well as poor sleep quality (r = −0.441, *p* = 0.027). A positive correlation was observed between the mentioned parameter and sleep efficiency (r = 0.439, *p* = 0.028). mRNA BDNF and mRNA TNF ratios pre/post therapy were also negatively correlated (r = −0.572, *p* = 0.004). Abbreviations: AIS—Athens Insomnia Scale, BDNF—Brain-Derived Neurotrophic Factor, TNF—Tumor Necrosis Factor, PSQI—Pittsburgh Sleep Quality Index.

Interestingly, mRNA BDNF ratio pre/post therapy was negatively correlated with the severity of insomnia symptoms (AIS score) at the baseline (r = −0.409, *p* = 0.042; Table 6; Figure 5a), poor sleep quality (r = −0.441, *p* = 0.027; Table 6; Figure 5b). 

Fittingly, a positive correlation was observed between the discussed ratio and sleep efficiency (r = 0.439, *p* = 0.028; Table 6; Figure 5c). mRNA BDNF and mRNA TNF ratios pre/post therapy were also negatively correlated (r = −0.572, *p* = 0.004; Table 7; Figure 5d). 

Differences between proBDNF and BDNF serum level observed before and after anti-TNF therapy did not reach statistical significance.

## 4. Discussion

The subject of neurotrophins in IBD patients has been scarcely studied. Available evidence suggests that the course of IBD might be heavily influenced by this protein group; p75NTR and TrkB are expressed in the gastrointestinal tract, the gut–brain axis allows for communication between the gut and the CNS, and the BDNF pathway is involved in regulation of the serotonergic signaling, which could be implicated in the pathophysiology of IBD [36]. Moreover, NTs have an important role in psychological conditions, and as data show, IBD patients face mental health issues more frequently than the general population. 

In the pilot study, it was observed that CD individuals have a higher level of BDNF protein concentration than HCs [27]. Current results are in line with those findings; IBD patients appear to have increased levels of proBDNF, BDNF, but not BDNF mRNA. It is important to note that BDNF can be produced in multiple compartments, one of them being peripheral blood mononuclear cells. It is probable that measuring both extra- and intracellular protein level would yield a statistically significant difference. Such discrepancy between protein concentration and gene expression could also stem from the fact that BDNF has several immature forms, and each stage involving modifications of BDNF precursors could be influenced by different factors. Interestingly, a positive correlation between proBDNF and BDNF levels was also noted, which implies that in IBD patients, the equilibrium between these two forms is maintained. These results are in line with the data obtained by other authors [37,38]. As Steinkampf et al. have demonstrated, inflammation might cause an increase in BDNF production in the enteric glial cells (EGC), the main source of NTs in the gut [37]. Moreover, EGCs also express TrkB receptors, which could suggest the existence of an autocrine loop, possibly disrupted in the course of the IBD [37]. However, the observed BDNF increase did not substantially decrease the EGCs’ apoptosis [37]. Similarly, in rats with colitis induced using dextran sodium sulfate, the expression of BDNF in longitudinal smooth muscle cells was higher compared to controls [38]. What is more, the administration of pro-inflammatory cytokines, TNF, and IL-1beta further enhanced BDNF production, which is opposite to our findings; in IBD subjects, baseline mRNA for BDNF and TNF were negatively correlated [24]. It is possible that peripheral BDNF and TNF mRNA expressions differ from those seen within the tissues. Fujiwara et al. have not observed any significant differences in serum BDNF level between the controls and the IBD patients [39]. However, their study group consisted, in the majority, of patients in clinical remission, many of whom were on biological maintenance therapy, and, thus, substantially differed from ours [39].

A negative correlation was observed between the BDNF protein concentration and the severity of depression symptoms assessed with BDI; the expression of BDNF mRNA was positively correlated to the questionnaire score in IBD subjects. In our earlier study, no correlation between BDNF protein concentration and BDI score in CD was seen, and the results obtained by other authors appear to align with those presented in this study [25,40,41].

Except for a correlation between BDNF and sleep efficiency in IBD subjects, none of the associations of measured sleep parameters (sleep latency, daytime sleepiness, sleep quality, total sleep time, symptoms of insomnia) was significant. This is unexpected, as other studies have found BDNF to be associated with sleep macrostructure and mentioned sleep qualities, insomnia in particular. A recent meta-analysis concluded that insomnia is associated with decreased BDNF level [42]. On the other hand, previously, we have observed a positive correlation between the severity of insomnia and the BDNF protein concentration [27]. Sleep quality was found to be negatively correlated with BDNF in female Japanese workers; however, no such differences were observed in a study on Brazilian adolescents [11]. In another study on subjects with obstructive sleep apnea (OSA), higher serum BDNF was associated with increased ESS score [43]. Moreover, OSA subjects with more pronounced insomnia symptoms and impaired sleep quality were shown to have lower evening level of serum BDNF and proBDNF [44]. However, as Santiago et al. found, not BDNF but cortisol awakening response bears a closer relation to sleep quality [45]. To conclude, studies evaluating the connection between BDNF and subjective sleep parameters vary highly depending on the population. Including objective sleep parameters as well as analysis of other psychological factors associated with BDNF, such as stress, would allow for a better insight.

Two types of IBD, UC and CD, differ in key clinical aspects regarding their symptoms, course, and treatment. In this study, the only difference between these groups was a higher BDNF protein concentration in UC than in CD; however, differences in BDNF mRNA and proBDNF were non-significant. This finding could be ascribed to a different group structure or sample size, rather than a specific UC feature, as no changes in the expression of BDNF mRNA were found. Results obtained by Fujiwara et al. are not in line with ours; the authors did not note any differences in BDNF protein concentration between UC and CD subjects; the expression of BDNF mRNA was not analyzed [39]. However, as already mentioned, their group was different from the one in this study [39].

In this study, there were no significant differences between IBD subjects in disease remission and exacerbation. Fujiwara et al., as well as our team in the preceding project, made similar observations [27,39]. However, in the present study, disease activity evaluation did not include endoscopy. Further investigation of this subject involving endoscopic assessment of disease severity, as well as immunohistochemical analysis of biopsy samples, would be desirable.

Biological therapy targeting TNF in IBD was proven safe and effective [26]. In this study, treatment with anti-TNF medications led to a decrease in TNF mRNA, while increasing BDNF expression. Protein concentrations were not affected. Of note, in many IBD participants, the baseline BDNF mRNA expression was close to null; therefore, such an increase in expression of this NT was rather rapid and unexpected. It needs to be further studied how long this enhanced BDNF production might last and whether it has any additional clinical significance beyond parameters studied here. BDNF mRNA pre- and post-ratio was also assessed, as it might reflect the dynamics of changes better than simple difference between relative mRNA expression level, taking into account variability in BDNF expression levels among individuals, additionally modified by physical activity, circadian rhythm, sex, etc. The discussed parameter was correlated negatively with poor sleep quality and severity of insomnia symptoms, but positively with sleep efficiency.

Similarly, we have previously observed no differences in BDNF protein concentration levels following biological anti-TNF therapy [25]. Studies on the subject of relation between anti-TNF therapy and BDNF vary. As was discussed above, BDNF transcription could be upregulated by pro-inflammatory cytokines. However, downstream modifications might rely on different factors, thus causing discrepancy between observed mRNA expression and protein concentration. In subjects with rheumatoid arthritis, therapy with infliximab decreased the BDNF level [46]. However, concentration of this protein was measured in plasma, not serum [46]. Regarding sleep quality, there is evidence that biological therapy improves sleep parameters, although those studies did not include BDNF and evaluation of its precursors’ levels [47,48].

A limitation of this study is the lack of endoscopic disease activity assessment. Instruments applied (partial Mayo score and HBI) were validated on the Polish population and are commonly used in clinical practice; however, there is no certainty that they would reflect alterations in the gastrointestinal tract caused by the disease. Sleep parameters in this study were also evaluated subjectively. This approach is advantageous in certain sleep disorders, such as in insomnia, which is diagnosed with sleep diaries and questionnaires instead of an objective method, such as actigraphy. Nevertheless, subjective estimation of sleep parameters by patients might be highly inaccurate. A combination of objective (i.e., actigraphy or polysomnography) and subjective methods would probably provide the most insight into sleep problems in IBD patients. Another problem commonly causing discrepancies in studies evaluating BDNF protein concentration is the use of either blood serum or plasma. Since BDNF is accumulated in platelets, differences in clotting time and centrifugation could affect the amount of BDNF released [49]. Some authors have also noted the lack of correlation between serum and plasma BDNF concentration [49]. Intracellular proBDNF and BDNF concentration also were not evaluated. It is possible that proteins produced and stored inside peripheral blood mononuclear cells could show a correlation with mRNA expression level and give more insight into differences between study groups.

An important asset of this study is the evaluation of three stages of BDNF production in different compartments. This approach takes into account the possibility that regulatory factors might vary depending on the level of this process. Moreover, proBDNF shows metabolic activity and can exert effects opposite to BDNF.

To conclude, differences in BDNF protein level observed between IBD patients and HCs might suggest that this NT is involved in the pathophysiology of IBD. As was observed in this and preceding studies, differences in protein concentration between certain groups of IBD patients are not consistent. Production of this neurotrophin is a multistage process, regulated by external and internal factors varying on an individual basis, such as age, sex, physical activity, or circadian rhythm [50]. Thus, protein concentration obtained from a single sample does not necessarily reflect the overall tendency in BDNF pathway activity. On the other hand, an mRNA expression level is less prone to alterations caused by the mentioned factors [51]. Thus, it could be inferred that measuring BDNF mRNA transcript might be a more reliable way of studying the activity of this pathway than protein concentration. Nevertheless, the latter method could be appropriate for a comparative analysis between the level of BDNF and proBDNF protein, as these two tend to exert an opposite effect on the metabolism. The association between the BDNF pathway, depression, and sleep should be further studied in IBD, as it seems to modulate the disease course [49]. Future studies on this subject might consider an analysis of other factors intertwined with BDNF, such as pain, physical activity, or stress. Due to the importance of BDNF to both somatic and psychological aspects of chronic inflammatory diseases, targeting this pathway in therapy might improve patients’ quality of life.

## Figures and Tables

**Table 1:** Study participants’ characteristics.

	IBD				
Parameter	All	Exacerbation	Remission	HC	*p*
n	80	47	33	44	-
Women (%, n)	56.2% (n = 45)	53.2% (n = 25)	60.6% (n = 20)	50% (n = 22)	0.504 ^a^ 0.510 ^b^
Age (median, IQR)	34.5 (28.0–41.0)	34.0 (29.0–41.0)	36 (25.0–42.0)	31.5 (25–45.5)	0.806 ^a^ 0.607 ^b^
BMI (kg/m^2^)	23.2 (20.6–25.8)	23.5 (20.8–27.1)	22.5 (19.7–25.0)	23.9 (20.6–26.8)	0.578 ^a^ 0.132 ^b^
Smoker (%, n)	13.8% (n = 11)	12.8% (n = 6)	15.2% (n = 5)	11.4% (n = 5)	0.786 ^a^ 0.754 ^b^
Chronic diseases (%, n)	23.8% (n = 19)	31.9% (n = 15)	12.1% (n = 4)	13.6% (n = 6)	0.243 ^a^ 0.061 ^b^
Steroids (%, n)	32.5% (n = 26)	51.1% (n = 24)	6.1% (n = 2)	0% (n = 0)	- 0.490 ^b^
Immunomodulators (%, n)	35.0% (n = 28)	31.9% (n = 15)	39.4% (n = 13)	0% (n = 0)	- *p* < 0.001 ^b^
Baseline TNF mRNA expression	78; 12.599 (6.03–47.59)	46; 10.951 (5.79–43.04)	32; 16.395 (7.01–57.75)	44; 16.491 (9.65–26.64)	0.817 ^a^ 0.222 ^b^
Years from diagnosis	-	8.0 (3.00–12.00)	7.0 (4.0–10.0)	-	- 0.496 ^b^
Surgery in the medical history	30.0% (n = 24)	31.9% (n = 15)	27.3% (n = 9)	25.0% (n = 11)	0.605 ^a^ 0.805 ^b^

^a^—IBD vs. HC. ^b^—Exacerbation vs. remission. Abbreviations: BMI—Body Mass Index, HC—Healthy Controls, IBD—Inflammatory Bowel Disease, TNF—Tumor Necrosis Factor, IQR—Inter-Quartile Range. Bolded text indicates statistically significant differences (*p* < 0.05). The term ‘chronic diseases’ indicates chronic non-inflammatory diseases, such as hypertension, hypercholesterolemia, type II diabetes, etc.

**Table 2:** Expression of neurotrophins in study’s participants.

	mRNA BDNF	proBDNF Protein	BDNF Protein
IBD	(n = 80); 655.70 (2.36–2850.57)	(n = 80); 14.69 (10.46–17.74)	(n = 80); 80; 25.36 (20.39–33.67)
HC	(n = 44); 1325.99(765.48–2261.07)	(n = 44); 11.56 (7.370–16.52)	(n = 44); 19.04(13.16–26.80)
*p*	**0.008**	**<0.001**	**0.008**
CD	(n = 49); 908.73 (17.06–3450.79)	(n = 49); 15.30(11.48–17.51)	(n = 49); 23.11(18.18–30.14)
UC	(n = 31); 306.07 (1.83–1830.11)	(n = 31); 13.31 (10.06–18.11)	(n = 31); 28.92 (23.65–37.89)
*p*	0.218	0.366	**0.007**
Ex	(n = 47); 605.81 (1.82–3088.55)	(n = 47); 14.71 (10.34–17.49)	(n = 47); 25.00 (20.05–33.16)
R	(n = 33); 705.60(2.81–1830.11)	(n = 33); 14.54 (10.86–18.47)	(n = 33); 27.37(21.00–34.17)
*p*	0.977	0.431	0.566
Ex	(n = 47); 605.81 (1.83–3088.55)	(n = 47); 14.71(10.34–17.49)	(n = 47); 25.00(20.05–33.16)
HC	(n = 44); 1325.99(765.48–2261.07)	(n = 44); 11.58 (7.37–16.52)	(n = 44); 19.04 (13.16–26.80)
*p*	**0.025**	**0.043**	**0.002**
R	(n = 33); 705.60 (2.81–1830.11)	(n = 33); 14.54(10.86–18.47)	(n = 33); 27.37(21.00–34.17)
HC	(n = 44); 1325.99(765.48–2261.07)	(n = 44); 11.58(7.37–16.52)	(n = 44); 19.04 (13.16–26.80)
*p*	**0.019**	**0.009**	**<0.001**

Abbreviations: BDNF—Brain-Derived Neurotrophic Factor, CD—Crohn’s Disease, Ex—Exacerbation, UC—Ulcerative Colitis, HC—Healthy Controls, IBD—Inflammatory Bowel Disease, proBDNF—BDNF precursor, R—Remission. Data are presented as n; median (IQR). Bolded text indicates statistically significant differences (*p* < 0.05).

**Table 3:** Correlations between level of expression of studied neurotrophins and selected sleep parameters.

	IBD		
	n	r	*p*
mRNA BDNF			
mRNA TNF	78	−0.76	**<0.001**
BDNF	80	−0.11	0.329
proBDNF	80	0.02	0.880
Sleep latency	80	0.03	0.799
Total sleep time	80	0.13	0.270
Total time spent in bed	80	0.12	0.278
Sleep efficiency [%]	80	0.09	0.441
BDNF protein			
mRNA TNF	78	0.17	0.150
proBDNF	80	0.29	**0.009**
Sleep latency	80	−0.13	0.266
Total sleep time	80	0.04	0.704
Total time spent in bed	80	−0.08	0.487
Sleep efficiency [%]	80	0.23	**0.037**
proBDNF protein			
mRNA TNF	78	0.07	0.519
Sleep latency	80	−0.03	0.802
Total sleep time	80	<0.00	0.995
Total time spent in bed	80	−0.15	0.185
Sleep efficiency [%]	80	0.15	0.190

Abbreviations: AIS—Athens Insomnia Scale, BDNF—Brain-Derived Neurotrophic Factor, IBD—Inflammatory Bowel Disease, proBDNF—BDNF precursor, PSQI—Pittsburgh Sleep Quality Index, TNF—Tumor Necrosis Factor. Data are presented as n; median (IQR). Bolded text indicates statistically significant differences (*p* < 0.05).

**Table 4:** Correlations between level of expression of studied neurotrophins and questionnaire scores.

	IBD		Exacerbation		Remission	
	r	*p*	r	*p*	r	*p*
mRNA BDNF						
AIS	0.06	0.619	−0.02	0.875	0.20	0.263
BDI	0.09	0.415	−0.11	0.463	0.36	**0.038**
ESS	0.04	0.715	0.04	0.780	0.04	0.821
PSQI	−0.01	0.936	−0.09	0.533	0.09	0.611
BDNF protein						
AIS	−0.04	0.760	−0.05	0.738	0.02	0.917
BDI	−0.20	0.078	−0.05	0.719	−0.35	**0.046**
ESS	0.09	0.425	0.13	0.396	0.08	0.674
PSQI	−0.12	0.310	−0.11	0.456	−0.04	0.836
proBDNF protein						
AIS	0.01	0.906	−0.01	0.956	0.09	0.640
BDI	−0.12	0.307	−0.03	0.822	−0.16	0.381
ESS	−0.08	0.504	0.20	0.263	−0.10	0.576
PSQI	0.04	0.759	0.17	0.267	−0.08	0.672

Abbreviations: AIS—Athens Insomnia Scale, BDI—Beck Depression Inventory, BDNF—Brain-Derived Neurotrophic Factor, ESS—Epworth Sleepiness Scale, IBD—Inflammatory Bowel Disease, proBDNF—BDNF precursor, PSQI—Pittsburg Sleep Quality Index. Bolded text indicates statistically significant differences (*p* < 0.05).

**Table 5:** Comparison between level of neurotrophin expression before and after anti-TNF therapy.

	Pre-Therapy	After 14 Weeks	*p*
mRNA BDNF	(n = 26); 151.13 (1.30–1604.28)	(n = 25); 1914.45 (585.17–4880.16)	**0.009**
mRNA TNF	(n = 25); 15.59 (7.05–49.44)	(n = 24); 7.84 (2.92–16.05)	**0.045**
BDNF protein	(n = 26); 22.81 (17.51–30.14)	(n = 25); 23.14 (16.01–29.20)	0.716
proBDNF protein	(n = 26); 13.25 (10.34–17.51)	(n = 26); 13.28 (10.54–17.59)	0.829

Abbreviations: BDNF—Brain-Derived Neurotrophic Factor, proBDNF—BDNF precursor, TNF—Tumor Necrosis Factor. Data are presented as n; median (IQR). Bolded text indicates statistically significant differences (*p* < 0.05).

**Table 6:** Correlations between changes in neurotrophin expressions post anti-TNF therapy, questionnaire scores, and sleep efficiency.

	IBD		
	n	r	*p*
mRNA BDNF ratio pre/post therapy			
BDI	25	−0.31	0.134
AIS	25	−0.41	**0.042**
ESS	25	−0.34	0.092
Sleep efficiency	25	0.44	**0.028**
PSQI	25	−0.44	**0.027**
BDNF protein ratio pre/post therapy			
BDI	24	0.45	0.658
AIS	24	0.23	0.288
ESS	24	0.06	0. 798
Sleep efficiency	24	−0.09	0.679
PSQI	24	0.11	0.579
proBDNF protein ratio pre/post therapy			
BDI	26	−0.07	0.749
AIS	26	−0.16	0.425
ESS	26	0.14	0.496
Sleep efficiency	26	0.32	0.111
PSQI	26	−0.04	0.839

Abbreviations: AIS—Athens Insomnia Scale, BDNF—Brain-Derived Neurotrophic Factor, BDI—Beck’s Depression Inventory, ESS—Epworth Sleepiness Scale, proBDNF—BDNF precursor, PSQI—Pittsburgh Sleep Quality Index. Bolded text indicates statistically significant differences (*p* < 0.05).

**Table 7:** Correlations between changes in neurotrophin expressions and tumor necrosis factor mRNA expression after biological treatment.

	IBD		
	n	r	*p*
mRNA BDNF ratio pre/post therapy and mRNA TNF ratio pre/post therapy	23	−0.57	0.004
BDNF ratio pre/post therapy and mRNA TNF ratio pre/post therapy	21	−0.19	0.420
proBDNF and mRNA TNF ratio pre/post therapy	23	−0.03	0.866

Abbreviations: BDNF—Brain-Derived Neurotrophic Factor, proBDNF—BDNF precursor, TNF—Tumor Necrosis Factor. Bolded text indicates statistically significant differences (*p* < 0.05).

## Data Availability

Data available on request from authors due to privacy restrictions.
